# Supplementary figures and images for: The First Genome-Wide Mildew Locus O Genes Characterization in the Lamiaceae Plant Family
Source: Int J Mol Sci. 2023 Sep 4;24(17):13627. doi: 10.3390/ijms241713627 (PMC10487521; doi:10.3390/ijms241713627)

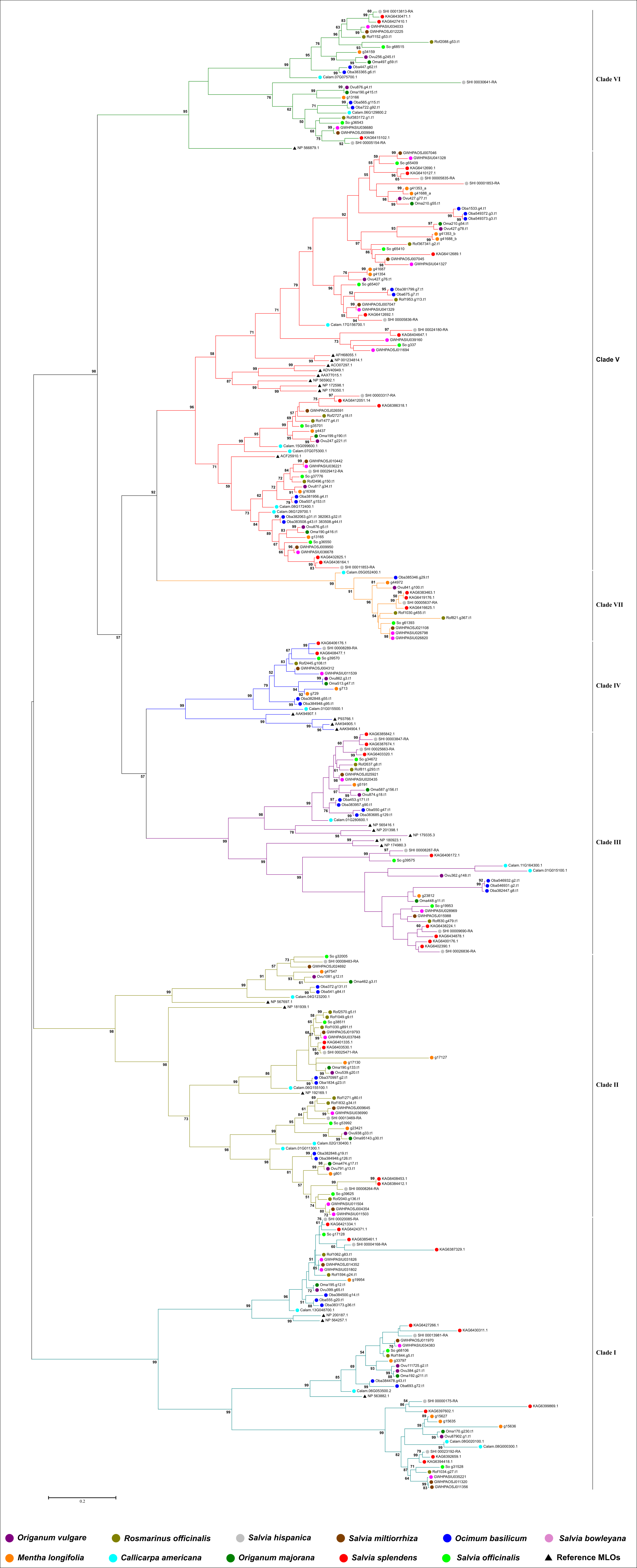

Supplement: Supplementary file 1 [file ijms-24-13627-s001.zip › Figure S1.png]
